# Allelic Complexity in Long QT Syndrome: A Family-Case Study

**DOI:** 10.3390/ijms18081633

**Published:** 2017-07-27

**Authors:** Alberto Zullo, Giulia Frisso, Nicola Detta, Berardo Sarubbi, Emanuele Romeo, Angela Cordella, Carlos G. Vanoye, Raffaele Calabrò, Alfred L. George, Francesco Salvatore

**Affiliations:** 1 CEINGE Biotecnologie Avanzate s.c.ar.l., 80145 Naples, Italy; zullo@ceinge.unina.it (A.Z.); gfrisso@unina.it (G.F.); detta@ceinge.unina.it (N.D.); cangela@unisa.it (A.C.); 2 Dipartimento di Scienze e Tecnologie, Università del Sannio, 82100 Benevento, Italy; 3 Dipartimento di Medicina Molecolare e Biotecnologie, Università di Napoli Federico II, 80131 Naples, Italy; 4 U.O.C. Cardiologia, A.O. Monaldi, Seconda Università di Napoli, 80131 Naples, Italy; berardo.sarubbi@virgilio.it (B.S.); ema.romeo@alice.it (E.R.); raffaele.calabro@unina2.it (R.C.); 5 Department of Pharmacology, Northwestern University Feinberg School of Medicine, Chicago, IL 60611, USA; carlos.vanoye@northwestern.edu; 6 IRCCS-Fondazione SDN, 80143 Naples, Italy

**Keywords:** long-QT syndrome, cardiac arrhythmias, potassium channels, electrophysiology, KCNQ1, KCNH2, HERG

## Abstract

Congenital long QT syndrome (LQTS) is associated with high genetic and allelic heterogeneity. In some cases, more than one genetic variant is identified in the same (compound heterozygosity) or different (digenic heterozygosity) genes, and subjects with multiple pathogenic mutations may have a more severe disease. Standard-of-care clinical genetic testing for this and other arrhythmia susceptibility syndromes improves the identification of complex genotypes. Therefore, it is important to distinguish between pathogenic mutations and benign rare variants. We identified four genetic variants (KCNQ1-p.R583H, KCNH2-p.C108Y, KCNH2-p.K897T, and KCNE1-p.G38S) in an LQTS family. On the basis of in silico analysis, clinical data from our family, and the evidence from previous studies, we analyzed two mutated channels, KCNQ1-p.R583H and KCNH2-p.C108Y, using the whole-cell patch clamp technique. We found that KCNQ1-p.R583H was not associated with a severe functional impairment, whereas KCNH2-p.C108Y, a novel variant, encoded a non-functional channel that exerts dominant-negative effects on the wild-type. Notably, the common variants KCNH2-p.K897T and KCNE1-p.G38S were previously reported to produce more severe phenotypes when combined with disease-causing alleles. Our results indicate that the novel KCNH2-C108Y variant can be a pathogenic LQTS mutation, whereas KCNQ1-p.R583H, KCNH2-p.K897T, and KCNE1-p.G38S could be LQTS modifiers.

## 1. Introduction

Long QT syndrome (LQTS) is an inherited predisposition to life-threatening ventricular arrhythmias that leads leading to sudden cardiac death in children and young adults [1], because prolongation of the QT interval is associated with delayed ventricular repolarization [2]. The prevalence of LQTS has recently been estimated to be ~1:2000 in Italian neonates [3]. LQTS can be inherited in an autosomal dominant fashion (Romano-Ward syndrome), which is the most frequent form, or as an autosomal recessive trait (Jervell and Lange-Nielsen syndrome), which is rare [4]. Sixteen different genes and more than 2000 mutations have been associated with congenital LQTS [5,6].

In most LQTS cases, causative mutations have been identified in the *KCNQ1* or *KCNH2* gene, which encode the pore-forming subunits of the two physiologically important potassium channels required for the slow and rapid delayed rectifier currents, *I*_Ks_ and *I*_Kr_, respectively [7,8]. Impaired function of these important repolarizing currents is the most common defect conferred by mutations in *KCNQ1* and *KCNH2* [9].

Given the substantial levels of genetic and allelic heterogeneity in LQTS, the existence of complex forms of the disease is not surprising. Between 4% and 11% of LQTS patients have more than one mutation in the same gene (i.e., compound heterozygosity) or have mutations in different *LQTS* genes (i.e., digenic heterozygosity) [10,11,12]. However, a single mutation can also induce the heterogeneous LQT phenotype, which is associated with differences in severity and clinical presentation, or overlapping syndromes [13]. LQTS subjects harboring two or more mutations may be more severely affected than their relatives who carry single mutations. With the emergence of genetic testing as the standard-of-care for LQTS, allelic complexity is likely to be observed more often, and strategies to resolve complex genotypes will become more important [5].

Incomplete penetrance is a very common feature of LQTS, and up to 40% of LQTS patients have normal QT intervals [14]. In our study, we identified four genetic variants in three genes (KCNQ1-p.R583H, KCNH2-p.C108Y, KCNH2-p.K897T, and KCNE1-p.G38S). These variants are independently assorted in five members of an Italian LQTS family in which symptoms occurred only in the offspring of healthy parents; therefore, no variant clearly segregated with the disease. The KCNQ1-p.R583H variant was previously reported to be associated with LQTS [15,16,17]; KCNH2-p.C108Y is a novel variant; and KCNH2-p.K897T and KCNE1-p.G38S were reported to influence the electrical activity of cardiac cells and to act as modifiers of the KCNH2 and KCNQ1 channels [9,18,19,20,21,22,23,24,25,26,27,28]. In order to better characterize the allelic complexity in our family, we performed in vitro heterologous functional expression studies.

## 2. Results

### 2.1. Clinical Phenotypes

**Figure 1:**
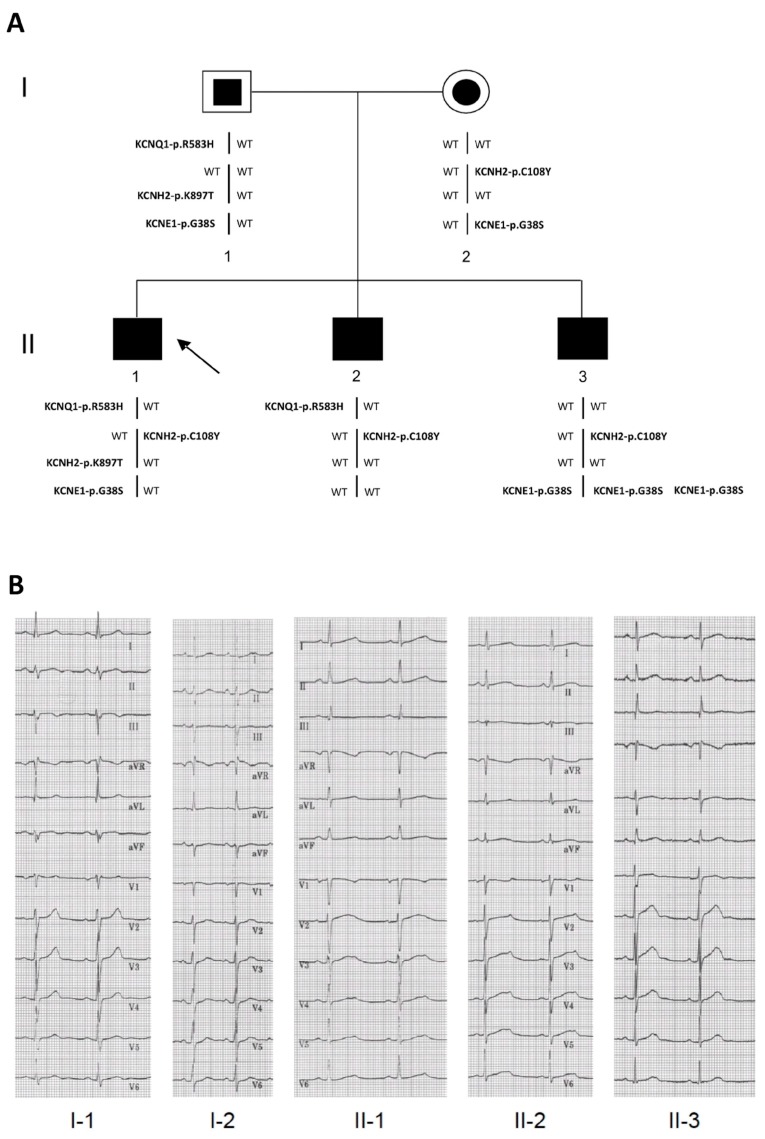
Pedigree and electrocardiograms. (**A**) Segregation of the KCNQ1-p.R583H, KCNH2-p.C108Y, KCNH2-p.K897T, and KCNE1-p.G38S variants in the long-QT syndrome (LQTS) family members. Black symbols indicate affected subjects with a mutant genotype; white symbols with a squared or circular black inset indicate unaffected subjects with a positive genotype; the black arrow indicates the proband. The karyotype of subject II-3 is 47, XY, +21; (**B**) Representative 12-lead electrocardiographic recordings from each member of the family.

Five members (three affected and two unaffected subjects) of a two-generation LQTS family from southern Italy were evaluated. The proband (II-1 in Figure 1A; Table 1) had syncope at 10 and 11 years of age, but cardiac evaluation showed no apparent anomalies. At age 12 years, the subject was found to have prolongation (560 ms) of the corrected QT interval (QTc) and started nadolol treatment, which resulted in only a mild reduction of the QTc interval. During the ensuing eight years of follow-up, the drug was changed to propranolol and no relevant clinical episodes occurred despite persistent prolongation of the QTc interval (Figure 1B).

A brother of the proband (II-2 in Figure 1A; Table 1) was evaluated by electrocardiography (ECG) during adolescence and was considered to have normal QT values. At age nine years he suffered an episode of syncope. After this episode, a complete cardiac evaluation revealed a long QTc (530 ms). He was then treated with nadolol and had a mild reduction of QTc (490 ms). Subsequently, his treatment was changed to propranolol and he was asymptomatic despite a prolonged QTc during the following 10 years (Figure 1B). Although asymptomatic, the other brother (II-3 in Figure 1A; Table 1) was screened because of the family history of LQTS and was found to have a QTc of ~470 ms at age six years. He was treated with nadolol, but three years after beginning treatment he experienced extreme bradycardia with sinus pauses of ~4 s followed by syncope. This prompted the placement of a dual (atrial and ventricular) pacing, dual (atrial and ventricular) sensing, dual response (inhibited and triggered) to sensing (DDD) pacemaker. During the next seven years, he was asymptomatic on propranolol and his QTc interval went back to the normal range (Figure 1B). Subject II-3 was also diagnosed with Down syndrome. Although neither genetically unrelated parent had cardiac symptoms, the father’s QTc values were at the upper limit of normal (QTc: 440 ms), and the mother’s were at the upper limit of borderline prolongation (QTc 471 ms, Figure 1B). Borderline LQTS is generally diagnosed when a patient has a QTc value between 440 and 470 ms [29].

### 2.2. Identification of the KCNQ1, KCNH2 and KCNE1 Variants

We identified four variants that could influence the length of the QT interval. Variant *KCNQ1*-c.G1748A results in the non-conservative substitution of arginine with histidine at position 583 (p.R583H) located within the C-terminal domain. Since this variant was previously reported in LQTS patients [15,16,17] and is rare in the general population (minor allele frequency (MAF) of 0.000016 in the Exome Aggregation Consortium (ExAC) database), it was initially considered a likely pathogenic mutation. Variant *KCNH2*-c.G323A causes the replacement of a cysteine residue by tyrosine (p.C108Y) within the N-terminal PAS (Per-Arnt-Sim) domain. Since KCNH2-p.C108Y had not previously been reported, we considered it to be a variant of uncertain clinical significance. Genetic screening of the *KCNH2* gene also revealed the *KCNH2*-c.A2690C (p.K897T) common variant (MAF = 0.187). This variant was previously reported to be a possible negative modifier of *I*_Kr_ that is able to alter channel kinetics, predispose to arrhythmic events, affect the repolarization process, and alter the length of the QT interval [9,18,19,20,21,22,23]. Another common variant in the *KCNE1* gene (c.G112A) results in the substitution of the glycine residue with serine at position 38 (p.G38S) and is located in the C-terminal cytoplasmic domain. The most updated MAF for this polymorphic variant is 0.352 (ExAC database). The *KCNE1*-c.G112A (p.G38S) variant has been reported to reduce KCNH2 and KCNQ1 channel currents, enhance KCNH2 susceptibility to QT-prolonging factors, and increase the risk for LQTS, atrial fibrillation, and heart failure [24,25,26,27,28].

Bioinformatic analysis predicted that KCNE1-G38S was “tolerated” and KCNH2-C108Y was “damaging”, whereas divergent results were obtained for KCNQ1-R583H and KCNH2-K897T, i.e., some programs considered these variants “damaging” and others as “benign” (Table 2). Moreover, the MAF of KCNQ1-p.R583H was much smaller (0.000016) than the estimated prevalence of LQTS (0.0005), whereas the MAFs of KCNH2-p.K897T and KCNE1-p.G38S were much larger (0.187 and 0.352, respectively). KCNH2-p.C108Y is not reported in the ExAC database.

All the known variants identified in the family were reported to have functional relevance for cardiac electrophysiology and, therefore, could potentially contribute to the pathogenesis of the LQTS phenotype in the family. As neither a variant nor a combination of variants clearly segregated with the LQTS phenotype, it was not possible to assign a priori pathogenicity to any of these variants without performing additional (e.g., functional) studies. Moreover, given the complex association of LQTS mutants in the individuals of the family and the potential causative role of each mutation, and also based on in silico analysis and on evidence from previous studies, we focused on the in vitro functional characterization of the KCNQ1-p.R583H and KCNH2-p.C108Y variants [9,18,19,20,21,22,23,24,25,26,27,28].

### 2.3. Functional Consequences of the KCNQ1-p.R583H and KCNH2-p.C108Y Variants

To investigate the functional consequences of KCNQ1-p.R583H and KCNH2-p.C108Y, we performed whole cell patch clamp experiments in transiently transfected CHO-K1 cells. KCNQ1-p.R583H mutant channels were investigated by analyzing ionic currents from cells expressing the wild-type (WT) channel (KCNQ1-WT) or KCNQ1-p.R583H and from cells co-expressing the KCNQ1 and KCNE1 subunits (KCNQ1-WT+KCNE1 or KCNQ1-p.R583H+KCNE1), which reconstitute the slow delayed rectifier potassium current (*I*_Ks_). The functional consequence of KCNH2-p.C108Y was investigated by analyzing the ionic currents from cells expressing KCNH2-WT or KCNH2-p.C108Y and from cells co-expressing both alleles.

#### 2.3.1. Functional Effects of KCNQ1-p.R583H

**Figure 2:**
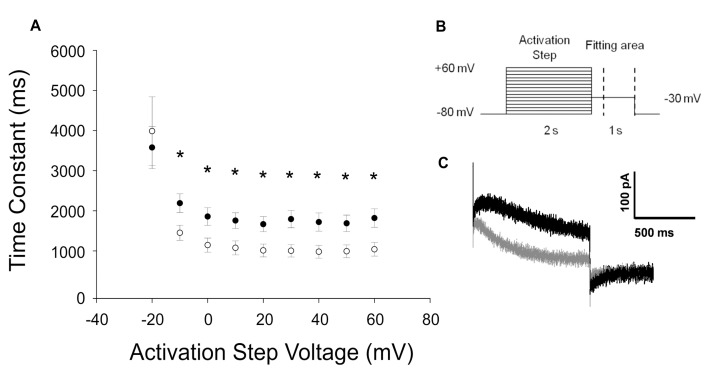
Functional characterization of the KCNQ1-p.R583H variant. (**A**) Time constants of the tail current decay of KCNQ1-WT (solid circles *n* = 13) and KCNQ1-p.R583H (open circles *n* = 11). Data were obtained by fitting the tail currents area, delimited by dashed lines shown in (**B**), with a single exponential function. Data are reported as mean ± standard error of the mean (SEM). * Significant differences between time constants from KCNQ1-WT and KCNQ1-p.R583H (*p* < 0.05); (**B**) Stimulation protocol; (**C**) Representative superimposed tail current traces recorded at −30 mV following +40 to +60 mV activating steps in cells transiently transfected with KCNQ1-WT (black trace) or with KCNQ1-p.R583H (gray trace).

The expression of KCNQ1-p.R583H in CHO-K1 cells evoked robust outward currents, and no significant differences in activating and tail current densities were identified between cells expressing a KCNQ1-p.R583H channel and cells expressing a KCNQ1-WT channel. However, there was a statistically significant positive shift in the voltage-dependence of activation (V_1/2_: KCNQ1-WT, −21.1 ± 0.86 mV, *n* = 13; KCNQ1-p.R583H, −16.7 ± 1.22 mV, *n* = 11, *p* < 0.05) without a significant alteration in the slope factor (k: KCNQ1-WT, 3.5 ± 0.7 mV, *n* = 13; KCNQ1-p.R583H, 4.6 ± 1.2 mV, *n* = 11, *p* > 0.05). In addition, the time course of deactivation was significantly faster in KCNQ1-p.R583H-expressing cells than in KCNQ1-WT-expressing cells (Figure 2). These findings indicate that the KCNQ1-p.R583H variant confers altered functional properties on the encoded channel.

**Figure 3:**
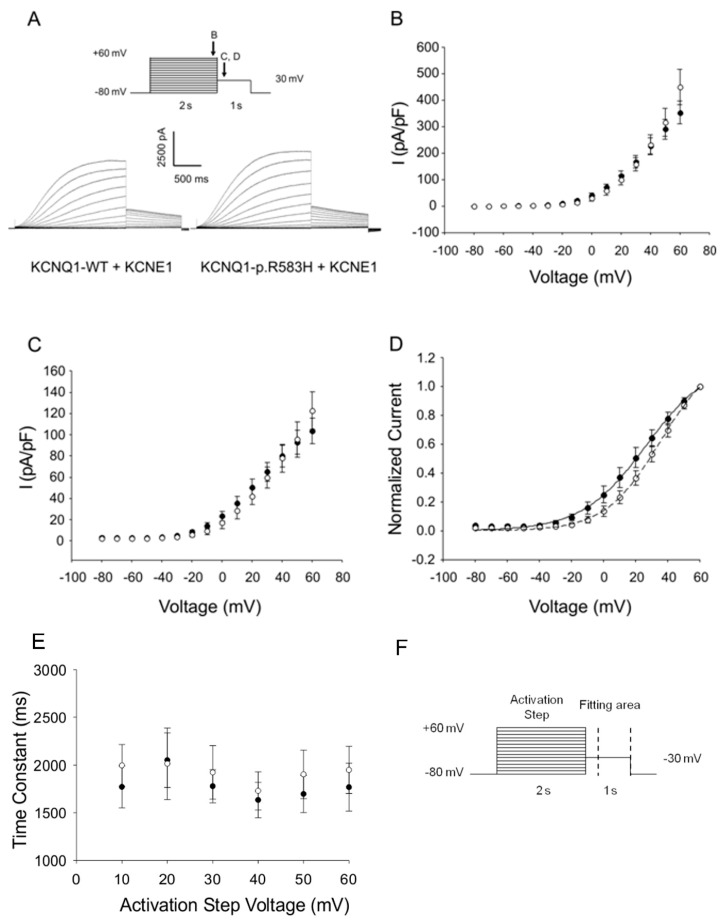
Functional characterization of the KCNQ1-p.R583H+KCNE1 complexes. (**A**) Representative traces illustrating the potassium currents observed in CHO-K1 cells transiently co-transfected with KCNQ1-WT+KCNE1 or KCNQ1-p.R583H+KCNE1 recorded with the protocol shown in inset (arrows indicate the time points at which currents were compared); (**B**) The current-voltage relation of potassium current densities (elicited by test pulses to various potentials and normalized to membrane capacitance) from CHO-K1 cells transiently transfected with KCNQ1-WT+KCNE1 (solid circles, *n* = 7) or KCNQ1-p.R583H+KCNE1 (open circles, *n* = 8); (**C**) The current-voltage relation of peak tail current densities after repolarization to −30 mV for KCNQ1-WT+KCNE1 (solid circles, *n* = 7) and KCNQ1-p.R583H+KCNE1 (open circles, *n* = 8); (**D**) Normalized current-voltage relation for peak tail current densities for KCNQ1-WT+KCNE1 (solid circles, *n* = 7) and KCNQ1-p.R583H+KCNE1 (open circles, *n* = 8). Data were recorded at test potentials ranging from −80 to +60 mV stepped in 10 mV increments from the holding potential of −80 mV for 2000 ms, followed by repolarization to −30 mV for 1000 ms. Data were fit with a Boltzmann distribution {*I* = *I*_max_/(1 + exp[(*V*_1/2_ − *V*)/*k*])} for KCNQ1-WT+KCNE1 (solid line) and for KCNQ1-p.R583H+KCNE1 (dashed line); (**E**) Time constants of the tail current from CHO-K1 cells transiently transfected with KCNQ1-WT+KCNE1 (solid line, *n* = 7) or KCNQ1-p.R583H+KCNE1 (open circles, *n* = 8) plotted as a function of the activation step voltage. The decay of the potassium current recorded during the test pulse to −30 mV was fit with a single exponential function (area delimited by dotted lines shown in **F**); (**F**) Stimulation protocol. Data are shown as mean ± SEM.

Since KCNQ1 channels associate with the accessory subunit KCNE1 to generate the slow delayed rectifier current (*I*_Ks_) [30,31], we performed additional co-expression studies to evaluate the functional consequences of KCNQ1-p.R583H on *I*_Ks_. The activating currents, tail currents, and voltage-dependence of the activation parameters did not differ between cells co-expressing KCNQ1-p.R583H+KCNE1 and cells co-expressing KCNQ1-WT+KCNE1 (*V*_1/2_: KCNQ1-WT+KCNE1, 31.8 ± 6.0 mV, *n* = 7; KCNQ1-p.R583H+KCNE1, 35.5 ± 5.9 mV, *n* = 8, *p* > 0.05; *k*: KCNQ1-WT+KCNE1, 16.86 ± 0.93 mV, *n* = 7; or KCNQ1-p.R583H+KCNE1, 15.38 ± 1.15 mV, *n* = 8, *p* > 0.05) (Figure 3). Based on these findings, we conclude that, unlike LQTS-associated mutations, the KCNQ1-p.R583H variant does not severely affect the function of the channel.

#### 2.3.2. KCNH2-p.C108Y Exhibits a Dominant-Negative Loss-of-Function

**Figure 4:**
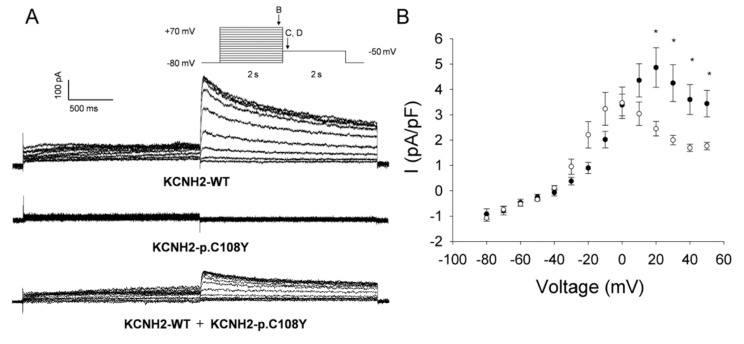

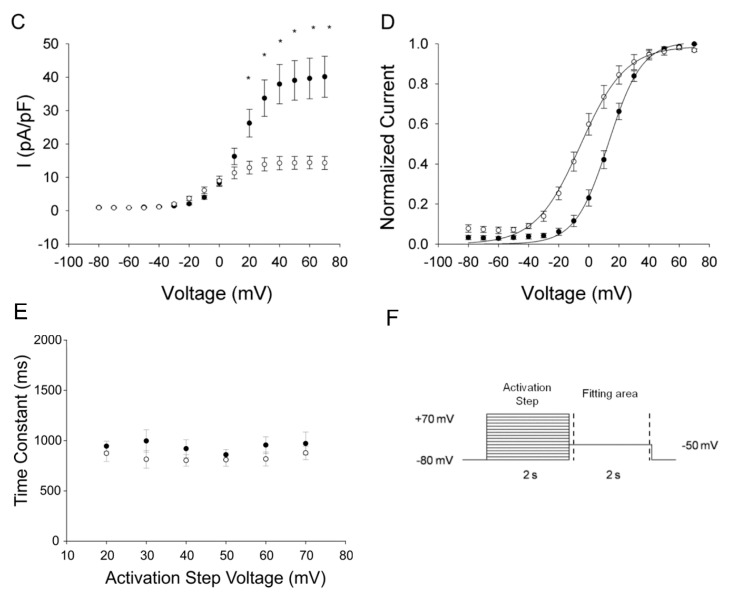
Functional properties of the KCNH2-p.C108Y variant. (**A**) Representative traces illustrating potassium currents recorded from CHO-K1 cells transiently transfected with KCNH2-WT and/or KCNH2-p.C108Y recorded with the protocol shown in the inset (arrows indicate the time points at which currents were compared); (**B**) Current-voltage relationships of potassium current densities (elicited by test pulses to various potentials and normalized to membrane capacitance) from CHO-K1 cells transiently transfected with KCNH2-WT+empty vector (solid circles, *n* = 6) or KCNH2-p.C108Y+KCNH2-WT (open circles, *n* = 9); (**C**) The current-voltage relation of the amplitude of the peak tail current densities after repolarization to −50 mV for KCNH2-WT+empty vector (solid circles, *n* = 6) and KCNH2-p.C108Y+KCNH2-WT (open circles, *n* = 9); (**D**) Normalized current-voltage relationships for peak tail current densities for KCNH2-WT+empty vector (solid circles, *n* = 6) and KCNH2-p.C108Y+KCNH2-WT (open circles, *n* = 9). Data were recorded at test potentials ranging from −80 to +70 mV in 10 mV from the holding potential of −80 mV for 2000 ms, followed by repolarization to −50 mV for 2000 ms. Data were fit with a Boltzmann distribution {*I* = *I*_max_/(1 + exp[(*V*_1/2_−*V*)/*k*])} for KCNH2-WT+empty vector (solid line) and for KCNH2-p.C108Y+KCNH2-WT (dashed line); (**E**) Time constants of the tail current from CHO-K1 cells transiently transfected with KCNH2-WT+empty vector (solid circles, *n* = 6) or KCNH2-p.C108Y+KCNH2-WT (open circles, *n* = 9) plotted as function of the activation step voltage. The decay of potassium current recorded during the test pulse to −50 mV was fit with a single exponential function (area delimited by dotted lines shown in **F**), representing the time constant for KCNH2 channel deactivation; (**F**) Stimulation protocol. Data are shown as mean ± SEM. * Significant differences between KCNH2-WT+empty vector and KCNH2-p.C108Y+KCNH2-WT (*p* < 0.05).

Heterologous expression studies demonstrated that KCNH2-p.C108Y is a non-functional channel (Figure 4A). To evaluate if this mutant channel could exert a dominant negative effect, we recorded whole-cell currents in CHO-K1 cells co-expressing KCNH2-WT and KCNH2-p.C108Y. Cells co-expressing KCNH2-WT and KCNH2-p.C108Y had significantly lower activating and tail current densities at activating potentials between +20 and +60 mV compared to cells co-transfected with KCNH2-WT and an empty plasmid vector (Figure 4B,C). In particular, the activating current density was ~50% lower and the tail current density was ~63% lower than in the KCNH2-WT control setting. Moreover, the voltage dependence of activation in cells co-expressing KCNH2-WT and KCNH2-p.C108Y was significantly shifted to more negative potentials compared to the control (V_1/2_: KCNH2-WT+empty vector, 12.9 ± 2.4 mV, *n* = 6; KCNH2-WT+KCNH2-p.C108Y, −7.0 ± 3.1 mV, *n* = 9; *p* < 0.05), but there were no differences in the slope factor (Figure 4D). We analyzed the deactivation of KCNH2-WT+KCNH2-p.C108Y complexes by fitting, with a single exponential function, the decay of the tail current at the −50 mV test pulse following the activation pulse (+20 to +70 mV) (Figure 4E,F). However, no significant differences in the deactivation process between KCNH2-WT+KCNH2-p.C108Y and KCNH2-WT were observed. These results indicate that KCNH2-p.C108Y confers a loss-of-function with dominant-negative features consistent with a pathogenic mutation causing autosomal dominant LQTS.

**Figure 5:**
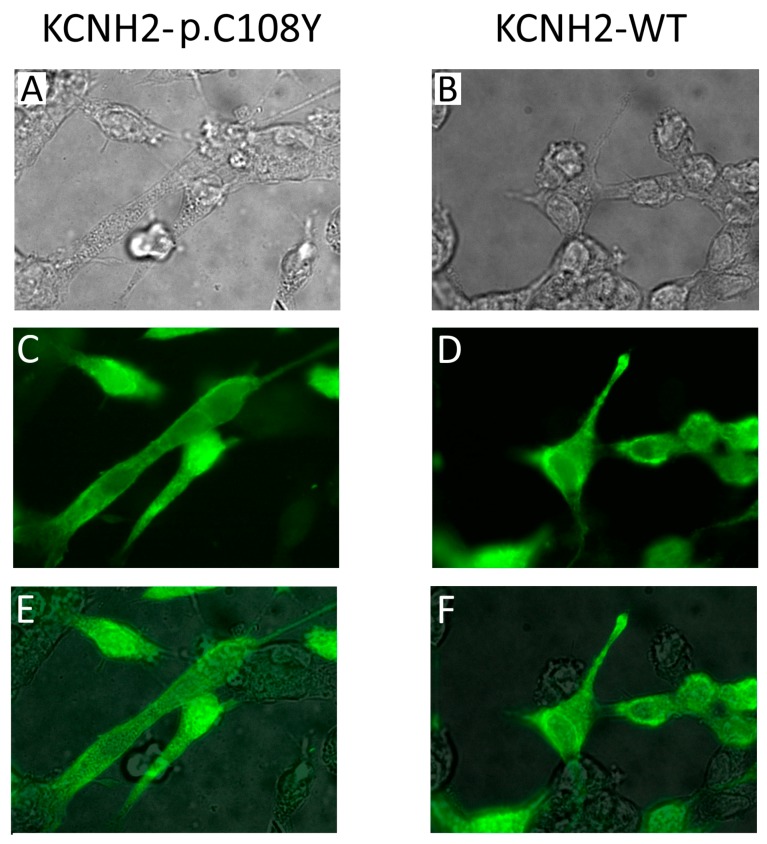
Immunocytochemical analysis of the KCNH2-p.C108Y variant. (**A**,**B**) 100× magnification phase-contrast images of HEK-293 cells transiently expressing KCNH2-p.C108Y or KCNH2-WT channels; (**C**,**D**) 100× magnification fluorescence images with cells immunolabeled for KCNH2 (green); (**E**,**F**) Merged phase-contrast and fluorescence images showing the localization of KCNH2 in the cells.

The C108Y substitution lies in the N-terminal PAS domain of the KCNH2 channel, the function of which is still not completely known. Nevertheless, it has been reported that mutations in this domain affect KCNH2 protein trafficking to the plasma membrane [32]. Therefore, we performed an immunocytochemical analysis on HEK-293 cells transiently expressing KCNH2-WT or KCNH2-p.C108Y (Figure 5). We found no significant difference between the immunofluorescence pattern of the cells transfected with KCNH2-WT and that of cells transfected with KCNH2-p.C108Y. These results do not support the hypothesis that KCNH2-p.C108Y is a trafficking-defective channel. Therefore, it is possible that this mutant could reach the membrane in either a mature or immature form, but its conductivity is compromised, possibly due to a defect in the domain-domain interaction function of PAS. Another mutation (KCNH2-p.C66G) with similar properties to the KCNH2-p.C108Y mutant has been described in the PAS domain of KCNH2, although in a different protein position [33]. In this case, the mutant channels localized on the plasma membrane are not in a mature form.

## 3. Discussion

Long QT syndrome is one the best characterized disorders among inherited arrhythmogenic syndromes. Screening of the five major genes involved in LQTS revealed one or more disease-causing mutations in about 70% to 80% and 4% to 9% of probands, respectively [16]. The presence of multiple mutations and single nucleotide polymorphism (SNPs) in *LQTS* genes can worsen the phenotype [16]. In our study, we analyzed a single LQTS family and identified, in three different genes, four sequence variants independently assorted in five family members. KCNH2-p.C108Y is an unknown variant. The KCNQ1-p.R583H variant is currently annotated as a mutation in the Human Gene Mutation Database (HGMD) database, having been identified in other LQTS subjects [15,16,17]. KCNH2-p.K897T was previously associated with a prolonged QT interval in several different populations and can alter the biophysical properties of mutant channels (current density, activation, inactivation, and recovery from inactivation) and exacerbate the *I*_Kr_ reduction caused by other KCNH2 mutations [18,19,20,21,22,23,34,35]. KCNH2-p.K897T affects also the synchronization between depolarization and repolarization and so increases the risk of cardiac mortality [18]. Therefore, it is a genetic modifier candidate. Finally, as reported in population studies, KCNE1-p.G38S is associated with heart failure, atrial fibrillation, abnormal cardiac repolarization, and an increased risk of ventricular arrhythmia [24,25,26,28]. Nevertheless, in vitro studies demonstrated that the KCNE1-p.G38S variant causes only a mild reduction of the delayed rectifier K+ currents [27]. Therefore, G38S could be a genetic modifier, but the evidence available does not suggest it has an overt effect on the function of the KCNQ1 and KCNH2 channels.

Given the complexity of the LQTS-related genetic background in our family, we functionally characterized only KCNH2-p.C108Y and KCNQ1-p.R583H. Our data demonstrate that the activity of KCNH2-p.C108Y was significantly lower than that of the wild type. This variant exerts a dominant-negative effect and shifts the voltage-dependence of activation when co-expressed with KCNH2-WT (Figure 4). Moreover, our data support the hypothesis that this variant, although localized in the PAS domain, which influences protein trafficking, can reach the plasma membrane. Therefore, we may speculate that the functional defect of KCNH2-C108Y could be compromised conductance. Interestingly, it has been reported that the KCNH2-p.C66G variant, located in the PAS domain, reaches the cell surface, but it remains in the immature form and is non-conducting [33]. On the contrary, the functionality of the KCNQ1-p.R583H channels was not severely compromised in a manner typical of LQTS-associated mutations.

Our study suggests that the KCNH2-p.C108Y variant has pathogenic properties consistent with LQTS. KCNH2-p.C108Y homozygous tetramers and KCNH2-WT/KCNH2-p.C108Y heterotetramers probably contribute less to the repolarizing current during action potentials and could affect the length of the QT interval. Moreover, the presence of other variants (KCNQ1-p.R583H, KCNH2-p.K897T, and KCNE1-p.G38S) could further enhance the effects of the mutant channels, thus resulting in incomplete penetrance and variable expressivity of the phenotype. On the contrary, in the mother, some other factors, including unknown genetic modifiers, could counteract the functional impairment of mutant channels, thereby protecting the asymptomatic KCNH2-p.C108Y mutation-positive subject from arrhythmia susceptibility.

We cannot rule out the presence, in this family, of other polymorphisms that alter the function of different ion channels. For example, SNPs in the *NOS1AP* gene have been associated with greatly deranged QT intervals in healthy subjects and in LQTS patients [36,37,38]. However, the risk stratification of LQTS patients does not include *NOS1AP* SNPs. Therefore, *NOS1AP* genotyping has not yet entered into clinical practice.

On the basis of the data collected in this study, we may speculate that the presence of KCNH2-p.C108Y, together with three KCNE1-p.G38S alleles, could lead to an increased risk of developing cardiac arrhythmias due to the prolongation of the QT interval. Moreover, the overt LQTS phenotype in our family could be caused by the co-expression, in cardiac cells, of KCNH2-p.C108Y and KCNQ1-p.R583H. The condition of digenic heterozygosity has been associated with a more severe phenotype, a higher risk of life-threatening events, and a reduced efficacy of beta blocking therapy [16]. Therefore, although our symptomatic patients do not exhibit a particularly aggressive phenotype, they were strongly advised to undergo close monitoring.

## 4. Materials and Methods

### 4.1. Clinical Investigations

Five members (three affected and two unaffected subjects) of a two-generation LQTS family were analyzed (Figure 1A). The three patients were referred to the Cardiology Division of the Monaldi Hospital (Naples, Italy) after diagnosis and treatment in other centers. Investigations included a complete medical history, physical examination, 12-lead standard ECG recordings, 24 h ECG Holter, and echocardiograms at various times during a period of at least 10 years. Patients were medically treated with beta-adrenergic receptor antagonists (β-blockers). Subject II-3 underwent invasive therapy (implantation of a DDD pacemaker) because of extreme bradycardia. The clinical data of the affected subjects are provided in Table 1.

### 4.2. Molecular Genetics

Informed consent for genetic analysis was obtained from the patients according to the procedure established by the Declaration of Helsinki, the Italian law, and the ethics committees of the participating institutions [39]. Genomic DNA was isolated from peripheral whole blood, as described elsewhere [40]. All exons including the 5′ and 3′ untranslated regions of the *KCNQ1*, *KCNH2*, *SCN5A*, *KCNE1*, and *KCNE2* genes were amplified by polymerase chain reaction (PCR). The PCR products were screened for mutations by direct sequencing (Applied Biosystems Big-Dye Terminator v1.1 cycle sequencing kit; Life Technologies, Grand Island, NY, USA) with an Applied Biosystems Prism 3100 DNA Analyzer (Life Technologies, Grand Island, NY, USA) using previously reported protocols [41]. In silico predictions of pathogenicity were made using the Alamut^®^ Visual software (Interactive Biosoftware, Rouen, France), and population allele frequencies were determined using the ExAC Browser (Beta) database [42,43].

### 4.3. Mutagenesis

Sequence variants *KCNH2*-c.G323A (p.C108Y) and *KCNQ1*-c.G1748A (p.R583H) were introduced into *KCNH2* and *KCNQ1* cDNAs, respectively, as described previously [44]. Primers used for mutagenesis are available upon request. The *KCNH2*-WT, *KCNQ1*-WT, and mutant coding sequences were engineered in bicistronic mammalian vectors pIRES2-EGFP (Biosciences-Clontech, Palo Alto, CA, USA). In addition, *KCNH2*-WT and *KCNE1*-WT were cloned in pIRES2-DsRed for co-expression experiments.

### 4.4. Cell Culture and Heterologous Expression

Chinese hamster ovaries (CHO-K1, American Type Culture Collection) were grown in F-12 HAM nutrient mixture medium (11765-054, ThermoFischer Scientific Inc., Waltham, MA, USA) supplemented with 10% fetal bovine serum (ThermoFisher Scientific Inc., Waltham, MA, USA), 2 mM l-glutamine, 50 U/mL penicillin, and 50 μg/mL streptomycin (Sigma, St. Louis, MO, USA) in a humidified 5% CO_2_ atmosphere at 37 °C. Cells were transfected using Fugene 6 (Roche, Mannheim, Germany) in 21 cm^2^ dishes with 1 μg (*KCNQ1*-WT or *KCNQ1*-c.G1748A) or 1.5 μg (*KCNH2*-WT or *KCNH2*-c.G323A) plasmid/dish in transient transfection experiments. For co-transfection experiments, 2 μg (1 μg *KCNQ1*-WT + 1 μg *KCNE1*-WT or 1 μg *KCNQ1*-c.G1748A + 1 μg *KCNE1*-WT) or 3 μg (1.5 μg *KCNH2*-WT + 1.5 μg *KCNH2*-c.G323A or 1.5 μg *KCNH2*-WT + 1.5 μg empty vector) plasmid per dish were used.

### 4.5. Cellular Electrophysiology

Electrophysiological experiments were carried out 48 and 72 h after transfection. Green fluorescent cells (in transient transfection experiments) and yellow fluorescent cells (in co-expression experiments) were selected for patch clamp recordings. Potassium currents were recorded at room temperature (22–24 °C) with the whole-cell patch clamp technique. Transfected CHO-K1 cells were plated on glass coverslips approximately 2 h before recording. Patch electrodes were pulled from standard wall borosilicate glass (G150F-3, Warner Instruments, Hamden, CT, USA) with a multistage P-97 Flaming-Brown micropipette puller (Sutter Instrument, San Rafael, CA, USA) and fire-polished with a Microforge MF-830 (Narishige, Tokyo, Japan). Electrode resistance ranged from 2.5 to 4 MΩ. The bath solution for *I*_HERG_ recording contained (in mM): 145 NaCl, 4 KCl, 1 MgCl_2_, 1.8 CaCl_2_, 10 4-(2-Hydroxyethyl)piperazine-1-ethanesulfonic acid (HEPES), and 10 glucose (pH 7.35, ≈310 mOsm/kg). The pipette solution contained (in mM): 110 KCl, 2 MgCl_2_, 10 Ethylenediaminetetraacetic acid, 10 HEPES, and 5 K_2_-ATP (pH 7.2, ≈255 mOsm/kg). The bath solution for *I*_KCNQ1_ and *I*_Ks_ experiments contained (in mM): 132 NaCl, 4.8 mM KCl, 1.2 mM MgCl_2_, 2 mM CaCl_2_, 10 mM HEPES, and 5 mM glucose (pH 7.4, ≈280 mOsm/kg). The pipette solution contained (in mM): 110 K-aspartate, 1 CaCl_2_, 1 MgCl_2_, 11 Ethylene glycol-*bis*(2-aminoethylether)-*N*,*N*,*N*′,*N*′-tetraacetic acid, 10 HEPES, 5 K_2_-ATP (pH 7.3, and ≈260 mOsm/kg). Pipette solutions were diluted 10% with distilled water to prevent swelling-activated currents. Chemicals were purchased from Sigma-Aldrich (St. Louis, MO, USA). A 2% agar-bridge composed of bath solution was used in the reference electrode. Data acquisition was carried out with an Axopatch 200 and 200B amplifier and pClamp 10.0 software (Molecular Devices, Corp., Sunnyvale, CA, USA). The junction potential was corrected and the series resistance was 90% compensated to minimize voltage errors. Whole-cell currents were filtered at 5 KHz, and the leak current was not subtracted. Specific voltage pulse protocols are fully described in the figures and legends.

### 4.6. Cell Culture and Immunocytochemistry

HEK-293 cells (American Type Culture Collection, Manassas, VA, USA) were grown in DMEM (11995065, ThermoFischer Scientific Inc., Waltham, MA, USA), supplemented with 10% fetal bovine serum (ThermoFisher Scientific Inc., Waltham, MA, USA), 2 mM l-glutamine, 50 U/mL penicillin, and 50 μg/mL streptomycin (Sigma-Aldrich, St. Louis, MO, USA) in a humidified 5% CO_2_ atmosphere at 37 °C. Cells grown on collagen-coated coverslips were transfected using Fugene 6 (Roche, Mannheim, Germany) with 1.5 μg (*KCNH2*-WT or *KCNH2*-c.G323A) plasmid/dish according to the manufacturer’s instructions. Forty-eight hours after transfection, cells were washed twice with phosphate-buffered saline (PBS) and fixed with 4% paraformaldehyde solution in PBS for 20 min. Cells were then washed with PBS, permeabilized with 0.2% triton X-100 solution in PBS for 5 min, and incubated in blocking solution (10% fetal bovine serum, 2% bovine serum albumin in PBS) for 1 h at room temperature. Samples were subsequently incubated with rabbit polyclonal anti-KCNH2 antibody (AB5908, Merck KGaA, Darmstadt, Germany; 1:200 dilution in blocking buffer) for 1 h at room temperature. After three washes with blocking solution, cells were incubated with fluorescein isothiocyanate-conjugated anti-rabbit immunoglobulin G (IgG) (heavy and light) antibody (AP307F, Merck KGaA, Darmstadt, Germany; 1:200 dilution in blocking buffer) and 4′,6-diamidino-2-phenylindole (D9542, Sigma-Aldrich, St. Louis, MO, USA; 10 mg/mL in blocking solution) for 1 h at room temperature. Unbound antibodies were washed off by blocking solution, and coverslips were mounted in 90% glycerol solution in PBS. Imaging was performed using an Axiovert 200 fluorescence inverted microscope (Zeiss, Oberkochen, Germany) equipped with a 100× Plan-Neofluar oil immersion objective (Zeiss, Oberkochen, Germany) and a CoolSNAP HQ2 CCD camera (Photometrics, Tucson, AZ, USA).

### 4.7. Data Analysis and Statistics

The cell membrane capacitance (*C*_m_) was calculated as a ratio of charge (estimated by integrating the area under the capacitive transients) to a 5 mV voltage pulse. We excluded from the analysis experiments showing *C*_m_ > 15 pF or series resistance >10 MΩ. The voltage-dependence of activation was obtained by fitting data with the Boltzmann function; *I* = *I*_max_/(1 + e[(*V*_1/2_ − *V*)/*k*]), with *I* = current at potential *V*, *I*_max_ = maximal normalized tail current, *V*_1/2_ = half-maximal activation voltage, and *k* = slope factor. The time course of deactivation was calculated by fitting tail currents with a single exponential function *I* = *A* e(−*t*/τ) + *B*, with *I* = current at time *t*, τ = time constant, *A*, *B* = amplitude constants. Data were collected for each experimental condition from at least three transient transfections and analyzed and plotted using Clampfit 10 (Molecular Devices, Corp., Sunnyvale, CA, USA) and SigmaPlot 11 (SPSS Science, Chicago, IL, USA). The standard two-tailed Student’s *t*-test (*p* < 0.05) was used to assess the statistical significance of inter-group differences in cases of normally distributed data according to the Kolmogorov-Smirnov test (*z*-value < 1.0). In other cases, the non-parametric Mann-Whitney test was used. The results are presented as mean ± standard error of the mean (SEM).

## 5. Conclusions

Our study demonstrates that a novel mutation, KCNH2-p.C108Y, dramatically impairs the I_Kr_ repolarizing current, whereas KCNQ1-p.R583H, although previously reported to cause LQTS, probably exerts sub-pathogenic defects that are more consistent with a modifier allele. Nevertheless, given the complex association of LQTS mutations in the individuals of the family examined, it was not possible to reconstruct all the combinations of the variants identified in the family, thus we were not able to perform a comprehensive in vitro functional characterization. Therefore, this study also highlights the limits of in vitro functional expression studies in the setting of LQTS. In fact, they can reveal the pathogenetic risk associated with variants of uncertain significance, but they are unable to resolve cases characterized by a high allelic complexity. Moreover, although heterologous expression systems can give important information about the activity of exogenous proteins, they are not the most appropriate environment for recapitulating the in vivo behavior of mutants. In order to completely resolve at functional level the allelic complexity observed in this family, the electrophysiological properties of cardiac cells should be evaluated in the light of the patient’s genotype. For this purpose, it would be advisable to perform an in vitro functional analysis on patient-specific cardiomyocytes derived from induced pluripotent stem cells.

## Figures and Tables

**Table 1:** Clinical data of the offspring of the long QT family.

Subjects *		II-1	II-2	II-3
Age at diagnosis		12 years	9 years	6 years
QTc ^†^	at diagnosis	560	530	470
	after therapy	470	470–490	420
HR	after therapy	56	58	80 ^#^
Syncope		Aged 10 years	Aged nine years	Aged 10 years, after 4 s sinus pause
		Aged 11 years		
Sinus pauses		No pause	No pause	Aged 9 years
				Aged 10 years
Therapy		Propanolol	Propanolol	Propanolol
				DDD-pacemaker
Other clinical information		None	None	Down syndrome

* The numbering of the subjects refers to the pedigree in Figure 1A; ^†^ QTc calculated by Bazett’s formula; ^#^ Programmed by dual (atrial and ventricular) pacing, dual (atrial and ventricular) sensing, dual response (inhibited and triggered) to sensing (DDD) pacemaker; HR: heart rate; QTc: corrected QT intrval; s: seconds.

**Table 2:** Analysis concerning variants in K-channels as predicted by bioinformatic tools.

Gene	Nucleotide Variation	Amino Acid Variation	MAF	Bioinformatic Tools	
				Polyphen	SIFT
*KCNE1*	G112A	G38S	0.352	Benign	Tolerated
*KCNH2*	G323A	C108Y	N.D.	Probably damaging	Damaging
*KCNH2*	A2690C	K897T	0.187	Benign	Not tolerated
*KCNQ1*	G1748A	R583H	0.000016	Benign	Not tolerated

N.D.: Not determined; MAF: Minor allele frequency.

## Abbreviations

Cm	Cell membrane capacitance
ECG	Electrocardiogram
HR	Heart rate
*I*_HERG_	Potassium current conducted by HERG channel
*I*_KCNQ1_	Potassium current conducted by KCNQ1 channel
*I*_Ks_	Slow delayed rectifier potassium current
*I*_Kr_	Rapid delayed rectifier potassium current
*k*	Slope factor
LQTS	Long QT syndrome
MAF	Minor allele frequency
mV	Millivolt
ms	Millisecond
MΩ	Megaohm
pA	Picoampere
pF	Picofarad
QTc	QT interval corrected for heart rate
s	Seconds
SEM	Standard error of the mean
*V*_1/2_	Half-maximal activation voltage
yrs	Years
